# Sodium-Glucose Cotransporter 2 Inhibitors and Risk of Retinopathy in Patients With Type 2 Diabetes

**DOI:** 10.1001/jamanetworkopen.2023.48431

**Published:** 2023-12-20

**Authors:** Fu-Shun Yen, James Cheng-Chung Wei, Teng-Shun Yu, Yu-Tung Hung, Chih-Cheng Hsu, Chii-Min Hwu

**Affiliations:** 1 Private practice, Taoyuan, Taiwan; 2 Department of Allergy, Immunology and Rheumatology, Chung Shan Medical University Hospital, Taichung City, Taiwan; 3 Institute of Medicine, Chung Shan Medical University, Taichung City, Taiwan; 4 Graduate Institute of Integrated Medicine, China Medical University, Taichung, Taiwan; 5 Management Office for Health Data, China Medical University Hospital, Taichung, Taiwan; 6 College of Medicine, China Medical University, Taichung, Taiwan; 7 Institute of Population Health Sciences, National Health Research Institutes, Zhunan, Miaoli County, Taiwan; 8 Department of Health Services Administration, China Medical University, Taichung, Taiwan; 9 Department of Family Medicine, Min-Sheng General Hospital, Taoyuan, Taiwan; 10 National Center for Geriatrics and Welfare Research, National Health Research Institutes, Yunlin County, Taiwan; 11 Section of Endocrinology and Metabolism, Department of Medicine, Taipei Veterans General Hospital, Taipei, Taiwan; 12 Faculty of Medicine, National Yang-Ming Chiao Tung University School of Medicine, Taipei, Taiwan

## Abstract

**Importance:**

Diabetic nephropathy and diabetic retinopathy share many similarities in pathophysiological processes. Preclinical studies have shown that sodium-glucose cotransporter 2 inhibitors (SGLT2is) have a protective role in the risk of diabetic retinopathy.

**Objective:**

To compare the risk of sight-threatening retinopathy associated with SGLT2is and other second-line glucose-lowering medications (including pioglitazone, sulfonylureas, and dipeptidyl peptidase-4 inhibitors [DPP-4is]) in patients with type 2 diabetes (T2D).

**Design, Setting, and Participants:**

This cohort study in Taiwan applied a new-user and active-comparator design. Patient demographic and clinical data were obtained from the National Health Insurance Research Database. Adult patients with newly diagnosed T2D from January 1, 2009, to December 31, 2019, were recruited and followed up until December 31, 2020. Propensity score matching was used to identify pairs of patients treated with SGLT2i vs DPP-4i, SGLT2i vs pioglitazone, and SGLT2i vs sulfonylurea from January 1, 2016, to December 31, 2019. Data were analyzed between August 18, 2022, and May 5, 2023.

**Exposures:**

Treatment with SGLT2i, DPP-4i, pioglitazone, and sulfonylureas starting on January 1, 2016.

**Main Outcomes and Measures:**

The main outcome was sight-threatening retinopathy in participants. Cox proportional hazards regression models were used to assess relative hazards of sight-threatening retinopathy between the matched case and control groups.

**Results:**

A total of 3 544 383 patients with newly diagnosed T2D were identified. After 1:1 propensity score matching, 65 930 pairs of patients treated with SGLT2i vs DPP-4i, 93 760 pairs treated with SGLT2i vs pioglitazone, and 42 121 pairs treated with SGLT2i vs sulfonylurea were identified. These matched patients included 236 574 males (58.6%), with a mean (SD) age of 56.9 (11.8) years. In the matched cohorts, SGLT2i had a significantly lower risk of sight-threatening retinopathy than DPP-4i (adjusted hazard ratio [AHR], 0.57; 95% CI, 0.51-0.63), pioglitazone (AHR, 0.75; 95% CI, 0.69-0.81), and sulfonylureas (AHR, 0.62; 95% CI, 0.53-0.71). The Kaplan-Meier curves showed that SGLT2i was associated with a significantly lower cumulative incidence of sight-threatening retinopathy than DPP-4i (3.52 vs 6.13; *P* < .001), pioglitazone (4.32 vs 5.76; *P* < .001), and sulfonylureas (2.94 vs 4.67; *P* < .001).

**Conclusions and Relevance:**

This cohort study found that SGLT2i was associated with a lower risk of sight-threatening retinopathy compared with DPP-4i, pioglitazone, and sulfonylureas. This finding suggests that SGLT2i may play a role not only in reduced risk of diabetic nephropathy but also in the slow progression of diabetic retinopathy in patients with T2D.

## Key Points

**Question:**

Could sodium-glucose cotransporter 2 inhibitors (SGLT2is) protect against the risk of sight-threatening diabetic retinopathy?

**Findings:**

In this cohort study of 3 544 383 patients with type 2 diabetes in Taiwan, SGLT2is were associated with a significantly lower risk and lower cumulative incidence of sight-threatening retinopathy than dipeptidyl peptidase-4 inhibitors, pioglitazone, and sulfonylureas.

**Meaning:**

Findings from this study suggest that SGLT2is may have an association not only with reduced risk of diabetic nephropathy but also with the slow progression of diabetic retinopathy in patients with type 2 diabetes.

## Teaser

This cohort study assesses the association of sodium-glucose cotransporter 2 inhibitors vs other second-line glucose-lowering medications with the development and incidence of diabetic retinopathy in adults with type 2 diabetes in Taiwan.

## Introduction

Sodium-glucose cotransporter 2 inhibitors (SGLT2is) can lower blood glucose levels, reduce body weight, and lower blood pressure by inhibiting proximal tubule glucose reabsorption and promoting urinary glucose excretion.^1^ A meta-analysis found that SGLT2is can effectively slow the progression of chronic kidney disease (CKD) by reducing renal hyperfiltration in patients with type 2 diabetes (T2D).^2^ However, there are associations and similarities between diabetic nephropathy and retinopathy.^3,4^ One study found that proteinuria and CKD are associated with the risk of diabetic retinopathy, and there is an association between diabetic retinopathy and the development or worsening of CKD.^3^ Chronic kidney disease and diabetic retinopathy share common risk factors, such as obesity, diabetes, and hypertension.^3^ Hyperglycemia can activate several biochemical pathways and promote oxidative stress accumulation in glomerular and retinal cells, resulting in chronic inflammation, cellular damage, endothelial dysfunction, and basement membrane thickening.^1,3,4^ Blood flow and circulation in the glomerulus and retina are regulated by microvasculature. Increased blood flow increases shear stress in glomerular and retinal capillaries, promoting inflammation and leakage.^3,4^ Studies have found that the embryogenetic stages in kidney and eye development occur concurrently, and renal and ocular organogenesis share several genes.^3,5^ Additionally, SGLT2is expressed in kidney mesangial cells and retinal pericytes may act as a glucose sensor to control cellular tone and regulation of blood flow.^4,6^ As CKD and retinopathy have several similarities in pathophysiological processes and SGLT2is can attenuate the development and progression of CKD, SGLT2is may also play a role in reducing the risk of diabetic retinopathy.^3,4^

Preclinical studies have found that dapagliflozin can decrease glucose uptake in human retinal endothelial cells, leading to less oxidative stress (retinal hydrogen peroxide as a marker) and inflammation (interleukin 6 as a marker).^4,7^ A study found that tofogliflozin in db/db mice can prevent the activation of glial fibrillary acidic protein and vascular endothelial growth factor (VEGF) production in the retina.^8^ Empagliflozin in Akimba mice can reduce vascular leakage and expression of VEGF in the retina.^9^ Phlorizin can attenuate pericyte swelling and normalize glucose uptake and type IV collagen overproduction in cultured bovine retinal pericytes.^6,10^ Dapagliflozin can reduce apoptosis in the diabetic retina and human retinal microvascular endothelial cells independent of hypoglycemic effects.^11^ Therefore, we hypothesized that SGLT2is play a protective role in the risk of diabetic retinopathy. We conducted a nationwide cohort study to compare the risk of sight-threatening retinopathy (including severe diabetic retinopathy and macular edema) associated with SGLT2is and other second-line glucose-lowering medications (including pioglitazone, sulfonylureas, and dipeptidyl peptidase-4 inhibitors [DPP-4is]) in patients with T2D.

## Methods

### Data Source 

The Taiwan government established the National Health Insurance (NHI) program in 1995. Under the NHI, the government is the sole purchaser and, along with employers, pays most of the insurance premium while the public pays only a small premium. Thus, by 2000, 99% of the 23 million residents in Taiwan were enrolled in the NHI. Patient data, such as sex, age, address, premium payments, examinations, diagnoses, prescriptions, and surgical procedures, are recorded in the NHI Research Database (NHIRD). Disease diagnoses are defined using *International Classification of Diseases, Ninth Revision (ICD-9)* and *International Statistical Classification of Diseases and Related Health Problems, Tenth Revision (ICD-10)* diagnostic codes. All clinicians caring for patients with diabetes, including general practitioners and ophthalmologists in private practice, report *ICD* codes to the NHIRD. The NHI administration conducts random and periodic inspections of medical records at clinics and hospitals throughout the country to improve the adequacy of disease management and the accuracy of diagnoses. This cohort study used the full population dataset of the NHIRD for patient identification and data analysis.^12^ All procedures were performed according to the Declaration of Helsinki.^13^ The China Medical University and Hospital Research Ethics Committee approved this study and waived the informed consent requirement because all identifiable clinician and patient data were scrambled and encrypted before database release. We followed the Strengthening the Reporting of Observational Studies in Epidemiology (STROBE) reporting guideline.

### Study Cohorts

We recruited patients with newly diagnosed T2D from January 1, 2009, to December 31, 2019, and followed up until December 31, 2020 (eFigure in Supplement 1). A diagnosis of T2D was defined as 1 hospitalization or at least 2 outpatient visits within 1 year using *ICD-9-CM (Clinical Modification)* code 250.xx, except 250.1x, and *ICD-10-CM* code E11 (eTable 1 in Supplement 1). The algorithm for the use of *ICD* diagnostic codes for the definition of T2D was validated by a previous Taiwanese study^14^ with acceptable accuracy.

We applied the new-user and active-comparator model for this cohort study. Generally, the model involves watching what happens when people try a drug for the first time and comparing the results with those of a well-established drug. This approach allowed us to ascertain whether the new drug was better, worse, or about the same as the usual drug. Patients treated with SGLT2i, DPP-4i, pioglitazone, or sulfonylureas for the first time after T2D diagnosis were defined as new users of those medications. For the SGLT2i, the index date was defined as the first date of SGLT2i use. Next, we calculated the duration (elapsed time) between the date of T2D diagnosis and the index date of SGLT2i. For pioglitazone, sulfonylureas, and DPP-4i, we identified the index date and elapsed time of use in the same way we defined those for SGLT2i. As SGLT2i has been marketed in Taiwan since May 2016, the index date for all drugs was set after January 1, 2016.

By observation, most patients were Han Taiwanese. Race and ethnicity were not available in the NHIRD or collected and analyzed for this study. Exclusion criteria were as follows: (1) age younger than 20 years or older than 80 years at first diagnosis of T2D, (2) missing information on sex or age, (3) diagnosis of type 1 diabetes or dialysis treatment before the index date, (4) study medication used within 1 year before the index date, and (5) diagnosis of sight-threatening retinopathy before the index date or death within 180 days after the index date to exclude potential mortality or morbidity due to other unrelated causes.

The clinically relevant variables used in the propensity score matching process were as follows (Table 1)^15^: age, sex, smoking status, comorbidities (obesity [composite of severely obese, obese, and overweight diagnoses], hypertension, coronary artery disease, stroke, heart failure, atrial fibrillation, peripheral artery disease, dyslipidemia, liver cirrhosis, chronic obstructive pulmonary disease, CKD, and diabetic retinopathy), Charlson Comorbidity Index (score range: 0 to ≥2, with the highest score indicating a greater burden of comorbid conditions and a higher risk of mortality),^16^ Diabetes Complications Severity Index (DCSI; score range: 0 to ≥2, with the highest score indicating a greater burden of diabetes complications),^17^ T2D diagnosis within 1 year prior to the index date, medications (sulfonylureas, metformin, α-glucosidase inhibitors, thiazolidinediones, and DPP-4i; number of oral antidiabetic agents; glucagon-like peptide-1 receptor agonists [GLP-1 RA]; insulin; aspirin; and statins), and duration of T2D.

**Table 1.** Baseline Characteristics of Matched Patients With Type 2 Diabetes Treated With Dipeptidyl Peptidase-4 Inhibitor (DPP-4i), Pioglitazone, Sulfonylurea, or Sodium-Glucose Cotransporter 2 Inhibitor (SGLT2i) Since 2016

Variable	Patient treatment, No. (%)		SMD^a^	Patient treatment, No. (%)		SMD^a^	Patient treatment, No. (%)		SMD^a^
	DPP-4i (n = 65 930)	SGLT2i (n = 65 930)		Pioglitazone (n = 93 760)	SGLT2i (n = 93 760)		Sulfonylurea (n = 42 121)	SGLT2i (n = 42 121)	
Sex									
Female	25 027 (38.0)	24 915 (37.8)	0.004	40 773 (43.5)	40 941 (43.7)	0.004	17 795 (42.2)	17 597 (41.8)	0.010
Male	40 903 (62.0)	41 015 (62.2)		52 987 (56.5)	52 819 (56.3)		24 326 (57.8)	24 524 (58.2)	
Age, y									
20-40	7405 (11.2)	7916 (12.0)	0.024	5593 (6.0)	5858 (6.3)	0.012	5198 (12.3)	5342 (12.7)	0.010
41-60	33 406 (50.7)	33 256 (50.4)	0.005	41 659 (44.4)	41 663 (44.4)	<0.001	20 812 (49.4)	21 014 (49.9)	0.010
61-80	25 119 (38.1)	24 758 (37.6)	0.011	46 508 (49.6)	46 239 (49.3)	0.006	16 111 (38.3)	15 765 (37.4)	0.017
Mean (SD) age^b^	55.9 (11.7)	55.6 (11.9)	0.019	59.5 (11.2)	59.3 (11.3)	0.011	55.8 (12.2)	55.5 (12.2)	0.020
Comorbidities									
Obesity	3748 (5.7)	4116 (6.2)	0.024	2416 (2.6)	2669 (2.9)	0.017	2783 (6.6)	3039 (7.2)	0.024
Smoking	3848 (5.8)	3792 (5.8)	0.004	4758 (5.1)	4731 (5.1)	0.001	2218 (5.3)	2210 (5.3)	0.001
Hypertension	45 019 (68.3)	45 154 (68.5)	0.004	67 173 (71.6)	67 036 (71.5)	0.003	28 927 (68.7)	28 763 (68.3)	0.008
Dyslipidemia	51 723 (78.5)	51 694 (78.4)	0.001	75 203 (80.2)	75 299 (80.3)	0.003	32 112 (76.2)	31 841 (75.6)	0.015
CAD	16 424 (24.9)	16 601 (25.2)	0.006	22 495 (24.0)	22 498 (24.0)	<0.001	11 543 (27.4)	11 702 (27.8)	0.008
Stroke	6069 (9.2)	6137 (9.3)	0.004	12 838 (13.7)	12 765 (13.6)	0.002	4478 (10.6)	4230 (10.0)	0.019
Heart failure	3264 (5.0)	3371 (5.1)	0.007	4375 (4.7)	4420 (4.7)	0.002	2502 (5.9)	2573 (6.1)	0.007
Atrial fibrillation	6197 (9.4)	6280 (9.5)	0.004	8910 (9.5)	8956 (9.6)	0.002	4785 (11.4)	4749 (11.3)	0.003
PAD	581 (0.9)	613 (0.9)	0.005	1193 (1.3)	1197 (1.3)	<0.001	385 (0.9)	374 (0.9)	0.003
COPD	15 552 (23.6)	15 558 (23.6)	<0.001	23 310 (24.9)	23 354 (24.9)	0.001	10 866 (25.8)	10 757 (25.5)	0.006
Liver cirrhosis	1158 (1.8)	1137 (1.7)	0.002	2443 (2.6)	2431 (2.6)	0.001	705 (1.7)	711 (1.7)	0.001
CKD	4739 (7.2)	4796 (7.3)	0.003	10 718 (11.4)	10 666 (11.4)	0.002	3251 (7.7)	3190 (7.6)	0.005
Diabetic retinopathy	5187 (7.9)	5156 (7.8)	0.002	10 052 (10.7)	9993 (10.7)	0.002	2477 (5.9)	2579 (6.1)	0.010
CCI score									
0	41 376 (62.8)	41 226 (62.5)	0.005	53 185 (56.7)	53 179 (56.7)	<0.001	26 873 (63.8)	26 746 (63.5)	0.006
1	12 718 (19.3)	12 576 (19.1)	0.005	18 222 (19.4)	18 290 (19.5)	0.002	7285 (17.3)	7440 (17.7)	0.010
≥2	11 836 (18.0)	12 128 (18.4)	0.011	22 353 (23.8)	22 291 (23.8)	0.002	7963 (18.9)	7935 (18.8)	0.002
DCSI score									
0	21 232 (32.2)	21 057 (31.9)	0.006	25 989 (27.7)	26 082 (27.8)	0.002	13 832 (32.8)	13 707 (32.5)	0.006
1	13 446 (20.4)	13 281 (20.1)	0.006	18 201 (19.4)	18 220 (19.4)	0.001	8206 (19.5)	8285 (19.7)	0.005
≥2	31 252 (47.4)	31 592 (47.9)	0.010	49 570 (52.9)	49 458 (52.8)	0.002	20 083 (47.7)	20 129 (47.8)	0.002
Medications									
Metformin	59 573 (90.4)	59 549 (90.3)	0.001	87 504 (93.3)	87 483 (93.3)	0.001	36 577 (86.8)	36 187 (85.9)	0.027
Sulfonylureas	39 722 (60.3)	39 652 (60.1)	0.002	71 578 (76.3)	71 574 (76.3)	<0.001	4042 (9.6)	4289 (10.2)	0.020
DPP-4i	13 205 (20.0)	13 101 (19.9)	0.004	51 526 (55.0)	51 434 (54.9)	0.002	15 889 (37.7)	16 188 (38.4)	0.015
α-Glucosidase inhibitors	10 955 (16.6)	10 991 (16.7)	0.001	24 787 (26.4)	24 716 (26.4)	0.002	5376 (12.8)	5472 (13.0)	0.007
No. of oral antidiabetic drugs									
0-1	23 637 (35.9)	23 684 (35.9)	0.001	15 011 (16.0)	14 897 (15.9)	0.003	23 410 (55.6)	23 216 (55.1)	0.009
2-3	38 328 (58.1)	38 289 (58.1)	0.001	62 057 (66.2)	62 251 (66.4)	0.004	17 968 (42.7)	18 058 (42.9)	0.004
>3	3965 (6.0)	3957 (6.0)	0.001	16 692 (17.8)	16 612 (17.7)	0.002	743 (1.8)	847 (2.0)	0.018
GLP-1 RA	5445 (8.3)	5495 (8.3)	0.003	23 266 (24.8)	23 274 (24.8)	<0.001	5359 (12.7)	5405 (12.8)	0.003
Insulin	23 904 (36.3)	23 914 (36.3)	<0.001	40 214 (42.9)	40 340 (43.0)	0.003	14 193 (33.7)	14 232 (33.8)	0.002
Statin	44 422 (67.4)	44 368 (67.3)	0.002	66 150 (70.6)	66 043 (70.4)	0.003	27 496 (65.3)	27 370 (65.0)	0.006
Aspirin	25 505 (38.7)	25 632 (38.9)	0.004	38 902 (41.5)	38 849 (41.4)	0.001	16 357 (38.8)	16 389 (38.9)	0.002
Duration of diabetes, mean (SD), y^b^	5.03 (3.6)	5.00 (3.6)	0.032	5.82 (3.3)	5.83 (3.3)	0.011	4.07 (3.4)	4.08 (3.4)	0.020

Abbreviations: CAD, coronary artery disease; CCI, Charlson Comorbidity Index; CKD, chronic kidney disease; COPD, chronic obstructive pulmonary disease; DCSI, Diabetes Complications Severity Index (score range: 0 to ≥2, with the highest score indicating a greater burden of diabetes complications); GLP-1 RA, glucagon-like peptide-1 receptor agonist; PAD, peripheral artery disease; SMD, standardized mean difference.

^a^ An SMD of 0.10 or lower indicated a negligible difference between SGLT2i, DPP-4i, pioglitazone, and sulfonylurea use.

^b^ Calculated with an unpaired, 2-tailed *t* test.

### Main Outcome 

The main outcome of this study was sight-threatening retinopathy in participants with at least 2 outpatient visits or 1 hospitalization for diabetic retinopathy and requiring surgery (NHI codes 86206B, 86207B, 86407B, and 86408B; *ICD-10-Procedure Coding System* [*PCS*] codes 08943ZZ, 08BE3ZZ, 08BF3ZZ, 08QE, and 08QF), laser photocoagulation (NHI codes 60001C, 60002C, 60005C, 60006C, 60003C, and 60004C; *ICD-10-PCS* codes 085E3ZZ and 08QE3ZZ), or anti-VEGF injections (ranibizumab, bevacizumab, or aflibercept) within 90 days of retinopathy diagnosis or vision loss (*ICD-9-CM* code 369; *ICD-10-CM* code H54).^15,18^ We followed up participants until sight-threatening retinopathy occurred or until the study ended on December 31, 2020, whichever occurred first.

### Statistical Analysis

To increase their comparability, we used 1:1 propensity score matching to balance the variables among participants treated with SGLT2i, DPP-4i, pioglitazone, and sulfonylurea.^19^ Considered as the case group in each comparison cohort, SGLT2i was selected repeatedly for matching with other non-SGLT2i treatments. Nonparsimonious multivariable logistic regression, with SGLT2i as the dependent variable, was used to estimate propensity scores for each patient. Twenty-nine clinically relevant variables, including baseline characteristics, comorbidities, medications, and duration of T2D, were used as independent variables, as listed in Table 1. We adopted the nearest-neighbor algorithm to construct matched pairs and assumed that the standardized mean difference (SMD) of 0.10 or lower between the matched case group and control group in each comparison cohort was negligible.

Conditional Cox proportional hazards regression models with robust sandwich SE estimates were used to compare the hazards of sight-threatening retinopathy between the matched case group and each control group. We used Schoenfeld residuals to test for the assumption of proportional hazards in the Cox proportional hazards regression models. Results were shown as hazard ratios and 95% CIs for SGLT2i vs DPP-4i, SGLT2i vs pioglitazone, or SGLT2i vs sulfonylurea. We used the Kaplan-Meier method to identify the cumulative incidence of sight-threatening retinopathy over time across the SGLT2i, DPP-4i, pioglitazone, and sulfonylurea treatments.

Subgroup analyses were performed to assess the risk of sight-threatening retinopathy in the age, sex, comorbidities, and medications variables between the matched SGLT2i cohort and DPP-4i, pioglitazone, or sulfonylurea cohorts. We conducted interaction tests to find the differential associations between subgroups of variables. For the subgroup analyses, we adjusted the significance threshold to a lower value (*P* < .005) to reduce the potential for type I error due to multiple comparisons.

We conducted a supplementary analysis to assess the risk of sight-threatening retinopathy associated with different SGLT2i treatments (empagliflozin, dapagliflozin, and canagliflozin) vs non-SGLT2i treatments to understand whether different SGLT2i treatments were associated with deviated outcomes. For secondary outcomes, we compared the hazards of dialysis, hospitalization for heart failure, and severe hypoglycemia (ie, patients who were referred to the emergency department or hospitalized for hypoglycemia) between the matched SGLT2i cohort and DPP-4i, pioglitazone, or sulfonylurea cohorts.

Statistical significance was defined as a 2-tailed *P* < .05 for the main analyses. Data were analyzed between August 18, 2022, and May 5, 2023, using SAS 9.4 (SAS Institute Inc).

## Results

The study identified from the NHIRD a total of 3 544 383 patients with newly diagnosed T2D from January 1, 2009, to December 31, 2019. After excluding ineligible patients, we identified 159 965 patients treated with SGLT2i, 304 383 treated with DPP-4i, 108 420 treated with pioglitazone, and 189 618 treated with sulfonylurea during the study period (eFigure in Supplement 1). We included the clinically related variables, such as age, sex, obesity, smoking, comorbidities, and medications, to match the 3 groups of patients with an SMD of 0.10 or lower, which was considered to be a negligible difference (Table 1). After 1:1 propensity score matching, we identified 65 930 pairs of patients treated with SGLT2i vs DPP-4i, 93 760 pairs treated with SGLT2i vs pioglitazone, and 42 121 pairs treated with SGLT2i vs sulfonylurea (eFigure in Supplement 1; Table 1). Of the overall matched patients, 167 048 were females (41.4%) and 236 574 were males (58.6%), with a mean (SD) age of 56.9 (11.8) years. The median (IQR) follow-up time in this study was 2.19 (1.53-3.16) years.

In the matched cohorts, the absolute numbers of sight-threatening retinopathy were 541 in the SGLT2i vs 928 in the DPP-4i groups, 1048 in the SGLT2i vs 1392 in the pioglitazone groups, and 284 in the SGLT2i vs 446 in the sulfonylurea groups. The multivariable-adjusted hazard ratios (AHRs) for sight-threatening retinopathy were 0.57 (95% CI, 0.51-0.63) for the SGLT2i vs DPP-4i, 0.75 (95% CI, 0.69-0.81) for the SGLT2i vs pioglitazone, and 0.62 (95% CI, 0.53-0.71) for the SGLT2i vs sulfonylurea groups (Table 2). The SGLT2i cohort had a significantly lower risk of the cumulative incidence of sight-threatening retinopathy than the DPP-4i (3.52 vs 6.13; *P* < .001), pioglitazone (4.32 vs 5.76; *P* < .001), and sulfonylureas cohorts (2.94 vs 4.67; *P* < .001) (Figure).

**Table 2.** Risks of Sight-Threatening Retinopathy in Matched Patients With Type 2 Diabetes

Risks of sight-threatening retinopathy	Patient treatment					
	SGLT2i	DPP-4i	SGLT2i	Pioglitazone	SGLT2i	Sulfonylureas
No. of events	541	928	1048	1392	284	446
Person-years	153 585	151 416	242 663	241 470	96 444	95 570
IR per 1000 person-years	3.52	6.13	4.32	5.76	2.94	4.67
Crude HR (95% CI)	0.58 (0.52-0.64)	1 [Reference]	0.75 (0.69-0.81)	1 [Reference]	0.63 (0.54-0.73)	1 [Reference]
AHR (95% CI)^a^	0.57 (0.51-0.63)	1 [Reference]	0.75 (0.69-0.81)	1 [Reference]	0.62 (0.53-0.71)	1 [Reference]
*P* value	<.001	NA	<.001	NA	<.001	NA

Abbreviations: AHR, adjusted hazard ratio; DPP-4i, dipeptidyl peptidase-4 inhibitor; HR, hazard ratio; IR, incidence rate; NA, not applicable; SGLT2i, sodium-glucose cotransporter 2 inhibitor.

^a^ Adjusted for age, sex, obesity, smoking, Charlson Comorbidity Index, Diabetes Complications Severity Index, comorbidities, medications, and duration of type 2 diabetes with the Cox proportional hazards regression model.

**Figure.**
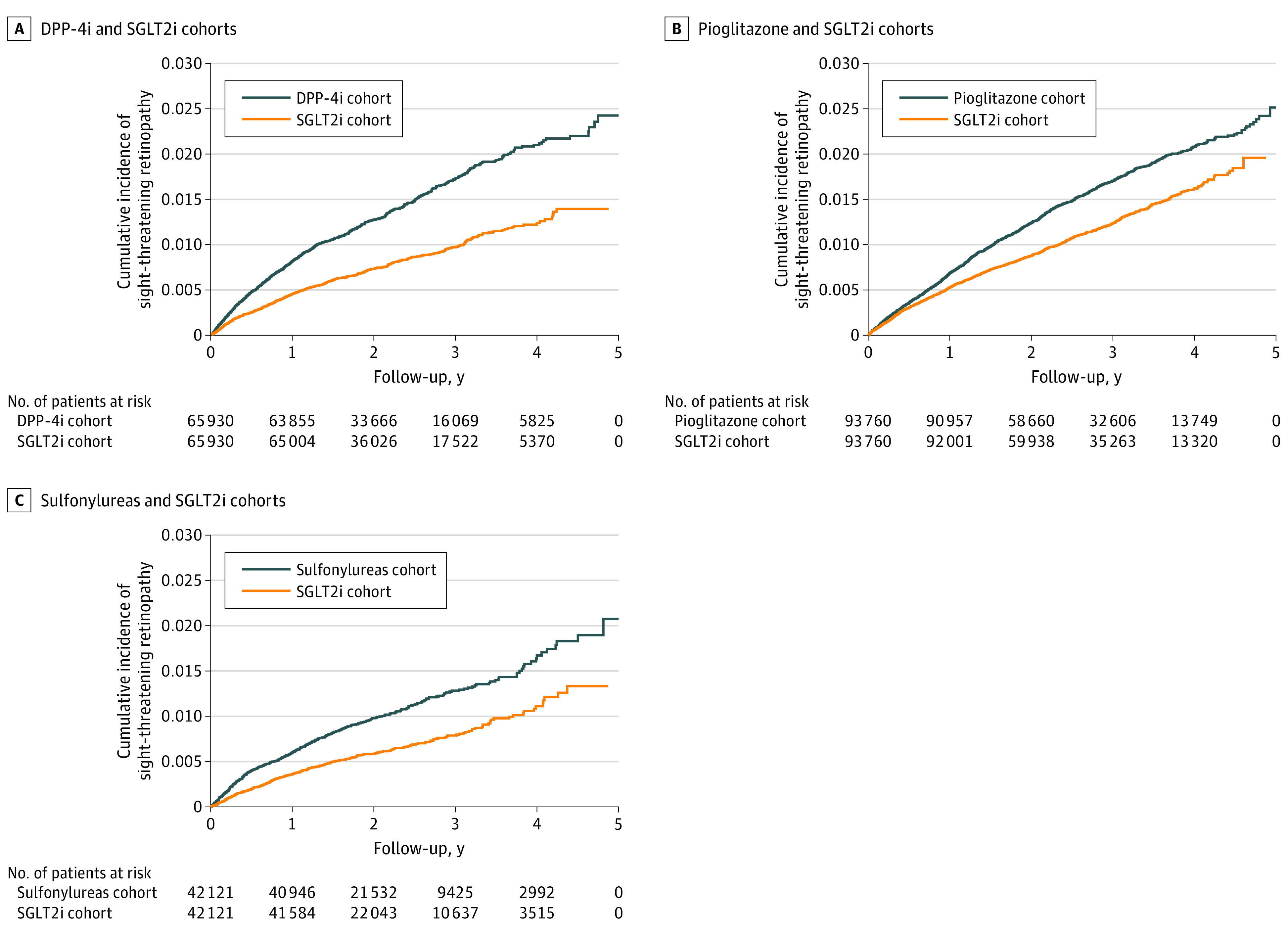
Cumulative Incidence of Sight-Threatening Retinopathy Between Medications DPP-4i indicates dipeptidyl peptidase-4 inhibitor; SGLT2i, sodium-glucose cotransporter 2 inhibitor.

### Subgroup and Additional Analyses

We found that SGLT2i was associated with a lower risk of sight-threatening retinopathy than DPP-4i in all subgroups of variables, with no significant interaction in the subgroup analyses (eTable 2 in Supplement 1). Additionally, SGLT2i was associated with a lower risk of sight-threatening retinopathy than pioglitazone in all subgroups of variables, and the interactions were significant in the metformin (AHR, 0.72; 95% CI, 0.67-0.79; *P* = .007), sulfonylurea (AHR, 0.71; 95% CI, 0.65-0.77; *P* = .009), and DPP-4i (AHR, 0.68; 95% CI, 0.61-0.75; *P* < .001) variable subgroups (eTable 3 in Supplement 1). Similarly, SGLT2i was associated with a lower risk of sight-threatening retinopathy than sulfonylureas in all subgroups of variables, and the interaction was significant in the insulin subgroup (AHR, 0.76; 95% CI, 0.61-0.94; *P* = .001) (eTable 4 in Supplement 1).

In matched patients, empagliflozin, dapagliflozin, and canagliflozin were associated with a significantly lower risk of sight-threatening retinopathy than DPP-4i, pioglitazone, and sulfonylureas (eg, vs DPP-4i: AHR, 0.53 [95% CI, 0.40-0.72]; 0.54 [95% CI, 0.48-0.62]; and 0.65 [95% CI, 0.57-0.74], respectively; *P* < .001) (eTable 5 in Supplement 1). Also in the matched cohorts, SGLT2i compared with DPP-4I, pioglitazone, and sulfonylurea was associated with a significantly lower risk of dialysis (eg, vs DPP-4i: AHR, 0.05; 95% CI, 0.03-0.08; *P* < .001), hospitalizations for heart failure (eg, vs DPP-4i: AHR, 0.47; 95% CI, 0.41-0.52; *P* < .001), and severe hypoglycemia (eg, vs DPP-4i: AHR, 0.44; 95% CI, 0.38-0.51; *P* < .001) (eTable 6 in Supplement 1).

## Discussion

This nationwide population-based cohort study found that SGLT2i was associated with a significantly lower risk of sight-threatening retinopathy than DPP-4i, pioglitazone, and sulfonylureas in patients with T2D. The results were consistent across the subgroups of age, sex, comorbidities, and medications.

The post hoc analysis of the EMPA-REG OUTCOME (Empagliflozin Cardiovascular Outcome Event Trial in Type 2 Diabetes Mellitus Patients–Removing Excess Glucose) trial found that empagliflozin was not associated with a significant difference in the risk of vision-threatening retinopathy compared with placebo.^20^ Three meta-analyses of randomized clinical trials also found that SGLT2i treatment was not associated with ocular events compared with no SGLT2i treatment.^21,22,23^ However, a meta-analysis found that SGLT2i was associated with a reduced risk of diabetic retinopathy in patients with diabetes for less than 10 years.^22^ Another meta-analysis reported that ertugliflozin and empagliflozin could reduce the risk of retinal disease, whereas canagliflozin could increase the risk of vitreous disease.^23^ The association of SGLT2i with decreased central retinal thickness and improved diabetic macular edema has been reported in several case reports.^8^ Su et al^24^ found that SGLT2i was associated with a significantly lower risk of diabetic macular edema compared with GLP-1 RA. The retrospective cohort by Lin et al^25^ found that SGLT2i compared with GLP-1 RA was associated with a lower risk of sight-threatening retinopathy but not the development of diabetic retinopathy. Dziuba et al^26^ used the Archimedes model to estimate 20-year cardiovascular and microvascular complications in patients with T2D. These investigators found that adding dapagliflozin to current treatment was associated with a 9.8% decrease in incident diabetic retinopathy compared with standard care.^26^ Findings from these clinical studies suggest that SGLT2i treatments are associated with a lower risk of diabetic retinopathy. The present study found that SGLT2i was associated with a significantly lower risk of sight-threatening retinopathy than DPP-4i, pioglitazone, and sulfonylureas in patients with T2D.

A small retrospective study of 82 patients with T2D found that DPP-4i treatment was associated with a significantly lower risk of diabetic retinopathy progression than non–DPP-4i treatment.^27^ However, a cohort study involving adults aged 65 years or older suggested that DPP-4i treatment lasting approximately 1 year was not associated with increased risk of diabetic retinopathy.^28^ A nationwide cohort study reported that DPP-4i add-on therapy was associated with a significantly higher risk of diabetic retinopathy progression than non–DPP-4i add-on therapy.^29^ A network meta-analysis found that DPP-4i was associated with a significantly higher risk of diabetic retinopathy events.^30^ A clinical cohort study revealed that SGLT2i was associated with a significantly lower risk of incident diabetic retinopathy but no significant difference in the risk of diabetic retinopathy progression compared with DPP-4i.^31^ These studies suggest that the association between DPP-4i and diabetic retinopathy is uncertain. In this study, we found that SGLT2i was associated with a significantly lower risk of sight-threatening retinopathy than DPP-4i in patients with T2D, and this result was consistent across different subgroups of patients.

A large cohort study involving 103 368 patients with T2D and without diabetic macular edema found that thiazolidinediones were associated with an increased risk of incident diabetic macular edema.^32^ A case-control study of 996 new cases of diabetic macular edema suggested that thiazolidinediones could play a role in diabetic macular edema development.^33^ A longitudinal study found an association between rosiglitazone and reduced risk of progression of diabetic retinopathy but without an association with diabetic macular edema.^34^ Subsequently, in a post hoc analysis of the ACCORD (Action to Control Cardiovascular Risk in Diabetes) eye study, 695 patients (20.0%) were treated with thiazolidinediones and 217 (6.2%) were diagnosed with macular edema.^35^ Thiazolidinediones could improve visual acuity and were not associated with an increased risk of diabetic macular edema.^35^ Results regarding the association of thiazolidinediones with diabetic retinopathy complications are conflicting. Sulfonylureas were associated with an increased risk of complications of diabetic retinopathy in a network meta-analysis of 36 clinical trials.^30^ A retrospective cohort study reported that SGLT2i treatment in patients with T2D could slow the progression of diabetic retinopathy compared with sulfonylureas.^36^ The present study found that SGLT2i was associated with a significantly lower risk of sight-threatening retinopathy than pioglitazone and sulfonylureas in patients with T2D.

There are several potential mechanisms by which SGLT2i was associated with a decreased risk of sight-threatening retinopathy. First, SGLT2i can lower blood glucose, blood pressure, uric acid, and body weight by increasing kidney excretion of glucose, sodium, and uric acid, thereby playing a role in reducing the risk of diabetic retinopathy, intracellular oxidative stress, visceral fat, and proinflammatory cytokines.^1,3,4^ Second, SGLT2i can reduce diabetic retinal and human microvascular endothelial cell apoptosis regardless of hypoglycemic effects.^11^ Third, SGLT2i can reduce pericyte swelling, increase microcirculation control, and normalize glucose uptake and type IV collagen overproduction in retinal pericytes.^6,10^ Fourth, SGLT2i can repair impaired retinal neurovascular coupling (including retinal blood flow dysregulation and neural retinal dysfunction) and inhibit retinal glial activation in mice with T2D.^8^ Fifth, animal studies have found that SGLT2i can reduce retinal vascular leakage and VEGF expression.^9^ Sixth, SGLT2i may downregulate the sympathetic nervous system and exert neuroprotection in the retina.^3,9,23^ In brief, SGLT2i has been associated with improved metabolism and microcirculation of the retinal neurovascular coupling and decreased apoptosis of retinal and microvascular endothelial cells to lower the risk of sight-threatening retinopathy.

### Strengths and Limitations

This study has some strengths. First, more than 95% of the population in Taiwan was enrolled in the NHI program, minimizing the possibility of selection bias. The number of patients with sight-threatening retinopathy was also higher, providing adequate power for performing subgroup analyses. Second, a period of 5 years (2016 to 2020) was covered, which was long enough for diabetic retinopathy to develop.^1,15^ Third, the new-user and active-comparator design was applied, which could restrict the potential for confounding by indication, reduce prevalent user bias, and allow a head-to-head comparison. Fourth, the clinical implication of this study was that SGLT2i treatments were as safe and effective in slowing the progression of diabetic retinopathy as in lowering the risk for diabetic nephropathy in patients with T2D.

This study also has several limitations. First, the NHIRD lacked information on participants’ family history, smoking status, alcohol consumption, and physical activity, which could have affected the results. Patients with suboptimal glycemic control or CKD were more prone to incident retinopathy and sight-threatening retinopathy. However, results of biochemical, blood glucose, hemoglobin A_1C_, and kidney function tests were unavailable in the NHIRD, preventing the assessment of participants’ diabetes management status and T2D severity. However, we matched CKD, diabetic retinopathy, and DCSI scores to balance the diabetes complications; we also matched the number of oral antidiabetic drugs, use of insulin, and duration of diabetes to balance the severity of T2D between the matched case and control groups. Second, the NHIRD lacked complete information on retinal fundoscopy, fluorescein angiography, and ocular computer tomography, which prevented the accurate identification of macular edema and diabetic retinopathy. However, we used *ICD* codes to identify baseline diabetic retinopathy and calculated DCSI scores to compare diabetes complications between the matched case and control groups. The algorithm of using *ICD-10-PCS* codes to define surgery for sight-threatening retinopathy has not been validated in previous studies, but because it is a clinical procedure performed by trained ophthalmologists, this algorithm might be more accurate than *ICD-9-CM* and *ICD-10-CM* codes. Third, the results may not apply to other races and ethnicities because the study participants were mainly Han Taiwanese in ethnicity. Fourth, a retrospective cohort study has residual confounding factors; therefore, the results can be interpreted only in terms of associations but not causation. Randomized clinical trials are warranted to confirm these results.

## Conclusions

This cohort study demonstrated that SGLT2i treatment was associated with a significantly lower risk of sight-threatening retinopathy than DPP-4i, pioglitazone, and sulfonylureas treatments in patients with T2D. The potential protective role of SGLT2i in sight-threatening retinopathy was observed in different subgroups of patients. In addition to playing a role in reducing the risk of diabetic nephropathy, SGLT2i may be associated with the slow progression of diabetic retinopathy.

## Notes

Supplement 1. **eTable 1.** Diseases and Associated ICD-9 and ICD-10 Codes **eTable 2.** Risk of Sight-Threatening Retinopathy in Patients With Type 2 Diabetes Treated With DPP-4i or SGLT2i Stratified by Variables **eTable 3.** Risk of Sight-Threatening Retinopathy in Patients With Type 2 Diabetes Treated With Pioglitazone or SGLT2i Stratified by Variables **eTable 4.** Risk of Sight-Threatening Retinopathy in Patients With Type 2 Diabetes Treated With Sulfonylureas or SGLT2i Stratified by Variables **eTable 5.** Risk of Sight-Threatening Retinopathy Associated With SGLT2i, DPP4i, Pioglitazone and Sulfonylureas Use **eTable 6.** Risk of Outcomes Between SGLT2i, DPP-4i, Pioglitazone and Sulfonylureas Use **eFigure.** Flowchart for the Selection of Matched Patients for SGLT2i, DPP-4i, Pioglitazone, and Sulfonylureas

Supplement 2.
Data Sharing Statement
