# Supplementary material for: Sodium-Glucose Cotransporter 2 Inhibitors and Risk of Retinopathy in Patients With Type 2 Diabetes
Source: JAMA Netw Open. 2023 Dec 20;6(12):e2348431. doi: 10.1001/jamanetworkopen.2023.48431 (PMC10733799; doi:10.1001/jamanetworkopen.2023.48431)
Supplement: Supplement 1. — eTable 1. Diseases and Associated ICD-9 and ICD-10 Codes eTable 2. Risk of Sight-Threatening Retinopathy in Patients With Type 2 Diabetes Treated With DPP-4i or SGLT2i Stratified by Variables eTable 3. Risk of Sight-Threatening Retinopathy in Patients With Type 2 Diabetes Treated With Pioglitazone or SGLT2i Stratified by Variables eTable 4. Risk of Sight-Threatening Retinopathy in Patients With Type 2 Diabetes Treated With Sulfonylureas or SGLT2i Stratified by Variables eTable 5. Risk of Sight-Threatening Retinopathy Associated With SGLT2i, DPP4i, Pioglitazone and Sulfonylureas Use eTable 6. Risk of Outcomes Between SGLT2i, DPP-4i, Pioglitazone and Sulfonylureas Use eFigure. Flowchart for the Selection of Matched Patients for SGLT2i, DPP-4i, Pioglitazone, and Sulfonylureas [file jamanetwopen-e2348431-s001.pdf]

Supplemental Online Content

Yen FS, Wei JCC, Yu TS, Hung YT, Hsu CC, Hwu CM. Sodium-glucose cotransporter 2 inhibitors and risk of retinopathy in patients with type 2 diabetes. *JAMA Netw Open*. 2023;6(12):e2348431. doi:10.1001/jamanetworkopen.2023.48431

eTable 1. Diseases and Associated ICD-9 and ICD-10 Codes

eTable 2. Risk of Sight-Threatening Retinopathy in Patients With Type 2 Diabetes Treated With DPP-4i or SGLT2i Stratified by Variables

eTable 3. Risk of Sight-Threatening Retinopathy in Patients With Type 2 Diabetes Treated With Pioglitazone or SGLT2i Stratified by Variables

eTable 4. Risk of Sight-Threatening Retinopathy in Patients With Type 2 Diabetes Treated With Sulfonylureas or SGLT2i Stratified by Variables

eTable 5. Risk of Sight-Threatening Retinopathy Associated With SGLT2i, DPP-4i, Pioglitazone and Sulfonylureas Use

eTable 6. Risk of Outcomes Between SGLT2i, DPP-4i, Pioglitazone and Sulfonylureas Use

eFigure. Flowchart for the Selection of Matched Patients for SGLT2i, DPP-4i, Pioglitazone, and Sulfonylureas

This supplemental material has been provided by the authors to give readers additional information about their work.

eTable 1. Diseases and Associated ICD-9 and ICD-10 Codes

Disease	ICD-9-CM codes	ICD-10-CM codes
Type 2 diabetes	250.xx, except 250.1x	E11
Type 1 diabetes	250.1x	E10
Dialysis	V56.0, V56.8, V45.1, V45.11	Z49.31, Z49.32, Z99.2
Obesity	278.02, 783.1, V85.2, 278.00, 649.1, V77.8, V85.3, 278.01, 649.2, V45.86, V85.4	R63.5, E66.09, E66.1, E66.8, E66.9, Z13.89, E66.01, E66.2
Smoking status	305.1, 649.0, V15.82	F17.200, F17.201, F17.210, F17.220, F17.221, F17.290, F17.291, Z87.891
Hypertension	401–405, A26	I10, I11, I12, I13, I15, N26
Dyslipidemia	272	E71.30, E71.31, E71.32, E71.39, E75.21, E75.22, E75.23, E75.24, E75.25, E75.29, E75.3, E75.4, E75.5, E75.6, E77, E78.0, E78.1, E78.2, E78.3, E78.4, E78.5, E78.6, E78.70, E78.71, E78.72, E78.79, E78.8, E78.9

© 2023 Yen F-S et al. *JAMA Network Open*

Coronary artery disease	398.91, 402, 404, 410-414, ICD-9-CM Procedure code (ICD-9-PCS): 36	I09.81, I11, I13, I20-I22, I24, I25.1-I25.7, I25.81, I25.89, ICD-10 Procedure code (ICD-10-PCS): 02
Stroke	430-438	G45.0, G45.1, G45.2, G45.3, G45.4, G45.8, G45.9, G46, I60, I61, I62, I63, I65, I66, I67.0, I67.1, I67.2, I67.3, I67.4, I67.5, I67.6, I67.7, I67.8, I67.9, I68, I69
Heart failure	428	I50
Atrial fibrillation	427	I45.0, I45.1, I45.2, I45.3, I45.4, I45.5, I45.6
Peripheral arterial disease	440.0, 440.20, 440.21, 440.22, 440.23, 440.24, 440.3, 440.4, 443.9, 443.81, 443.89	I70.2, I70.92, I75.0, I73.9
Chronic obstructive pulmonary disease	491, 492, or 496	J41, J42, J44, J43, or J44.9
Liver cirrhosis	571.5, 571.2, 571.6	K70.2, K70.30, K70.31, K74.0, K74.1, K74.2,

© 2023 Yen F-S et al. *JAMA Network Open*

		K74.60, K74.69, K74.3, K74.4, K74.5
Chronic kidney disease	250.4, 403-404, 585-586, 581.8, 593.9, 791.0, V42.0, V45.1, V56.0, V56.8 , ICD-9-PCS: 39.27, 39.42-39.43, 39.49-39.50, 39.53, 39.93-39.95	I12, I13, N08, N18, N19, N29, E10.2, E11.2, E13.2, N02.8, N04.7, N04.8, N18.9, N28.9, R80.8, R80.9, N18.1-N18.3, R80.0-R80.3, Z94.0, Z99.2, Z94.0, E10.65, E11.65, Z49.31, Z49.32
Diabetic retinopathy	362.02, 362.07, 362.0	H35.0, E08.311-E08.359, E09.311-E09.359, E11.311-E11.359, E13.311-E13.359
Vision loss	369	H54
Hypoglycemia	251.0x, 251.1x, or 251.2x	E15, E16, E16.1, E16.2

eTable 2. Risk of Sight-threatening Retinopathy in Patients With Type 2 Diabetes Treated With DPP-4i or SGLT2i Stratified by Variables

Variables	With DPP-4i			With SGLT2i							
	N	PY	IR	N	PY	IR	cHR	95% CI	aHR[a]	95% CI	P for interaction
Sex											0.944
female	348*	57344	6.07	203	58753	3.46	0.57	(0.48, 0.68)	0.56	(0.47, 0.66)	
male	580	94073	6.17	338	94832	3.56	0.58	(0.51, 0.66)	0.57	(0.50, 0.66)	
Age											0.133
20-40	96	17624	5.45	46	18914	2.43	0.45	(0.32, 0.64)	0.46	(0.32, 0.66)	
41-60	487	77995	6.24	272	78721	3.46	0.55	(0.48, 0.64)	0.55	(0.47, 0.63)	
61-80	345	55798	6.18	223	55950	3.99	0.64	(0.54, 0.76)	0.64	(0.54, 0.76)	
Comorbidities											
Obesity	21	8787	2.39	20	9983	2.00	0.87	(0.47, 1.60)	0.97	(0.52, 1.82)	0.216
Smoking	34	8423	4.04	15	8281	1.81	0.45	(0.24, 0.82)	0.43	(0.23, 0.79)	0.411
Hypertension	632	103568	6.10	372	105045	3.54	0.58	(0.51, 0.66)	0.58	(0.51, 0.66)	0.815
Dyslipidemia	632	119689	5.28	364	121061	3.01	0.57	(0.50, 0.65)	0.56	(0.49, 0.64)	0.829
Coronary artery disease	157	38064	4.12	116	38192	3.04	0.73	(0.57, 0.93)	0.75	(0.59, 0.95)	0.029
Stroke	82	13550	6.05	43	14004	3.07	0.51	(0.35, 0.73)	0.53	(0.37, 0.77)	0.488
Heart failure	39	7237	5.39	27	7424	3.64	0.67	(0.41, 1.09)	0.66	(0.40, 1.09)	0.544
Atrial fibrillation	58	13885	4.18	34	14016	2.43	0.57	(0.38, 0.88)	0.60	(0.39, 0.92)	0.983

© 2023 Yen F-S et al. *JAMA Network Open*

Peripheral arterial disease	9	1350	6.67	8	1431	5.59	0.83	(0.32, 2.15)	0.71	(0.25, 1.97)	0.436
COPD	147	34983	4.20	102	35405	2.88	0.68	(0.53, 0.88)	0.67	(0.52, 0.86)	0.136
Liver cirrhosis	11	2301	4.78	10	2459	4.07	0.84	(0.36, 1.98)	0.74	(0.29, 1.88)	0.353
Chronic kidney disease	84	10338	8.13	45	10518	4.28	0.54	(0.37, 0.77)	0.54	(0.37, 0.78)	0.597
Diabetic retinopathy	175	12134	14.42	101	12827	7.87	0.56	(0.44, 0.71)	0.54	(0.43, 0.7)	0.750
CCI											0.971
0	526	96307	5.46	306	96676	3.17	0.58	(0.50, 0.67)	0.56	(0.48, 0.64)	
1	192	29804	6.44	108	29687	3.64	0.57	(0.45, 0.72)	0.56	(0.44, 0.71)	
≥2	210	25305	8.30	127	27223	4.67	0.56	(0.45, 0.70)	0.59	(0.47, 0.73)	
DCSI											0.305
0	273	48992	5.57	139	49207	2.82	0.51	(0.41, 0.62)	0.51	(0.42, 0.63)	
1	170	31220	5.45	110	31514	3.49	0.64	(0.50, 0.82)	0.63	(0.49, 0.8)	
≥2	485	71204	6.81	292	72864	4.01	0.59	(0.51, 0.68)	0.58	(0.50, 0.67)	
Medications											
Metformin	843	137566	6.13	485	139955	3.47	0.57	(0.51, 0.63)	0.56	(0.50, 0.62)	0.383
Sulfonylureas	699	93691	7.46	381	95660	3.98	0.54	(0.47, 0.61)	0.53	(0.47, 0.60)	0.031
Thiazolidinediones	273	31376	8.70	142	32664	4.35	0.5	(0.41, 0.62)	0.49	(0.40, 0.60)	0.139
Alpha-glucosidase inhibitors	173	26154	6.61	123	27250	4.51	0.69	(0.54, 0.86)	0.68	(0.54, 0.86)	0.090
Numbers of oral antidiabetic drugs											0.661
0-1	207	51903	3.99	129	51767	2.49	0.62	(0.50, 0.78)	0.62	(0.50, 0.78)	
2-3	636	89927	7.07	366	91829	3.99	0.57	(0.50, 0.64)	0.56	(0.49, 0.63)	

© 2023 Yen F-S et al. *JAMA Network Open*

>3	85	9586	8.87	46	9989	4.60	0.52	(0.36, 0.75)	0.52	(0.36, 0.74)	
GLP-1 RA	93	12015	7.74	44	12544	3.51	0.45	(0.32, 0.65)	0.46	(0.32, 0.66)	0.184
Insulin	465	54179	8.58	275	56550	4.86	0.57	(0.49, 0.66)	0.56	(0.48, 0.65)	0.955
Statin	570	102915	5.54	309	104614	2.95	0.53	(0.46, 0.61)	0.53	(0.46, 0.60)	0.098
Aspirin	322	58888	5.47	203	59593	3.41	0.62	(0.52, 0.74)	0.62	(0.52, 0.74)	0.280

PY, Person-Year; IR, incidence rate, per 1000 person-years; cHR, crude hazard ratio; aHR, adjusted hazard ratio; CI, confidence interval; COPD, chronic obstructive pulmonary disease; SGLT2i, sodium-glucose cotransporter-2 inhibitor; DPP-4i, dipeptidyl peptidase-4 inhibitor; GLP-1 RA, glucagon-like peptide-1 receptor agonist; CCI, Charlson Comorbidity Index; DCSI, Diabetes Complications Severity Index; aHR[a]: adjusted for age, sex, obesity, smoking, Charlson Comorbidity Index, Diabetes Complications Severity Index score, comorbidities, medications, and duration of diabetes as shown in Table 1 with the Cox proportional hazards regression.

* This means that during the 57344 patient-years of follow-up in the matched female DPP-4i users, 348 women developed sight-threatening retinopathy, with an incident rate of 6.07 (per 1000 PY).

© 2023 Yen F-S et al. *JAMA Network Open*

eTable 3. Risk of Sight-threatening Retinopathy in Patients With Type 2 Diabetes Treated With Pioglitazone or SGLT2i Stratified by Variables

Variables	With pioglitazone			With SGLT2i							
	N	PY	IR	N	PY	IR	cHR	95% CI	aHR[a]	95% CI	P for interaction
Sex											0.381
female	586*	105292	5.57	432	108441	3.98	0.72	(0.64, 0.81)	0.70	(0.62, 0.79)	
male	806	136178	5.92	616	134222	4.59	0.77	(0.70, 0.86)	0.78	(0.70, 0.86)	
Age											0.466
20-40	74	15136	4.89	64	15916	4.02	0.85	(0.61, 1.19)	0.85	(0.61, 1.20)	
41-60	645	110397	5.84	459	110614	4.15	0.71	(0.63, 0.80)	0.69	(0.62, 0.78)	
61-80	673	115937	5.80	525	116134	4.52	0.78	(0.69, 0.87)	0.78	(0.70, 0.88)	

© 2023 Yen F-S et al. *JAMA Network Open*

Comorbidities											
Obesity	23	6387	3.60	16	7247	2.21	0.61	(0.32, 1.15)	0.63	(0.33, 1.21)	0.542
Smoking	52	11446	4.54	23	11356	2.03	0.44	(0.27, 0.72)	0.45	(0.27, 0.74)	0.035
Hypertension	988	173133	5.71	741	173742	4.26	0.75	(0.68, 0.82)	0.74	(0.67, 0.82)	0.928
Dyslipidemia	1007	194113	5.19	771	195669	3.94	0.76	(0.69, 0.84)	0.75	(0.68, 0.82)	0.596
Coronary artery disease	273	58302	4.68	203	58244	3.49	0.74	(0.62, 0.89)	0.75	(0.62, 0.9)	0.933
Stroke	197	31769	6.20	157	32373	4.85	0.79	(0.64, 0.97)	0.81	(0.65, 1.00)	0.645
Heart failure	63	10858	5.80	52	10929	4.76	0.83	(0.57, 1.20)	0.84	(0.58, 1.23)	0.635
Atrial fibrillation	94	22598	4.16	77	22500	3.42	0.82	(0.61, 1.11)	0.82	(0.61, 1.11)	0.534
Peripheral arterial disease	27	3126	8.64	16	3021	5.30	0.61	(0.33, 1.13)	0.64	(0.34, 1.20)	0.509
COPD	263	58720	4.48	202	58953	3.43	0.77	(0.64, 0.92)	0.75	(0.63, 0.91)	0.804
Liver cirrhosis	26	5542	4.69	24	5711	4.20	0.91	(0.52, 1.59)	0.92	(0.52, 1.62)	0.517

© 2023 Yen F-S et al. *JAMA Network Open*

Chronic kidney disease	186	25669	7.25	117	25026	4.68	0.64	(0.51, 0.81)	0.67	(0.53, 0.85)	0.157
Diabetic retinopathy	301	25589	11.76	228	26943	8.46	0.73	(0.62, 0.87)	0.72	(0.61, 0.86)	0.734
CCI											0.123
0	696	140093	4.97	561	139091	4.03	0.81	(0.72, 0.90)	0.78	(0.69, 0.87)	
1	295	47643	6.19	202	48134	4.20	0.68	(0.57, 0.82)	0.70	(0.58, 0.83)	
≥2	401	53734	7.46	285	55438	5.14	0.69	(0.60, 0.81)	0.72	(0.61, 0.84)	
DCSI											0.070
0	323	68253	4.73	226	67833	3.33	0.71	(0.60, 0.84)	0.71	(0.59, 0.84)	
1	226	47768	4.73	208	48110	4.32	0.91	(0.75, 1.10)	0.87	(0.72, 1.05)	
≥2	843	125449	6.72	614	126720	4.85	0.72	(0.65, 0.80)	0.72	(0.65, 0.80)	
Medication											
Metformin	1311	225901	5.80	963	228338	4.22	0.73	(0.67, 0.79)	0.72	(0.67, 0.79)	0.007

© 2023 Yen F-S et al. *JAMA Network Open*

Sulfonylureas	1177	186152	6.32	851	189486	4.49	0.71	(0.65, 0.78)	0.71	(0.65, 0.77)	0.009
DPP-4i	885	130816	6.77	617	138691	4.45	0.66	(0.60, 0.74)	0.68	(0.61, 0.75)	<0.001
Alpha-glucosidase inhibitors	367	64383	5.70	325	67467	4.82	0.85	(0.73, 0.98)	0.84	(0.72, 0.97)	0.044
Numbers of oral antidiabetic drugs											0.023
0-1	145	37908	3.83	134	34795	3.85	0.97	(0.77, 1.23)	0.96	(0.75, 1.21)	
2-3	969	160803	6.03	682	161634	4.22	0.70	(0.64, 0.77)	0.70	(0.63, 0.77)	
>3	278	42759	6.50	232	46234	5.02	0.78	(0.65, 0.93)	0.78	(0.65, 0.93)	
GLP-1 RA	354	55594	6.37	259	57637	4.49	0.71	(0.61, 0.84)	0.71	(0.61, 0.84)	0.453
Insulin	736	100768	7.30	571	104946	5.44	0.75	(0.67, 0.84)	0.75	(0.67, 0.84)	0.879
Statin	888	170759	5.20	661	172882	3.82	0.74	(0.67, 0.81)	0.72	(0.66, 0.80)	0.555
Aspirin	542	99677	5.44	426	100634	4.23	0.78	(0.69, 0.89)	0.78	(0.69, 0.89)	0.435

PY, Person-Year; IR, incidence rate, per 1000 person-years; cHR, crude hazard ratio; aHR, adjusted hazard ratio; CI, confidence interval; COPD, chronic obstructive pulmonary disease; SGLT2i, sodium-glucose cotransporter 2 inhibitor; DPP-4i, dipeptidyl peptidase-4 inhibitor;

© 2023 Yen F-S et al. *JAMA Network Open*

GLP-1 RA, glucagon-like peptide-1 receptor agonist; CCI, Charlson Comorbidity Index; DCSI, Diabetes Complications Severity Index; aHR[a]: adjusted for age, sex, obesity, smoking, Charlson Comorbidity Index, Diabetes Complications Severity Index score, comorbidities, medications, and duration of diabetes as shown in table 1 with the Cox proportional hazards regression.

* This means that during the 105292 patient-years of follow-up in the matched female pioglitazone users, 586 women developed sight-threatening retinopathy, with an incident rate of 5.57 (per 1000 PY).

© 2023 Yen F-S et al. *JAMA Network Open*

eTable 4. Risk of Sight-threatening Retinopathy in Patients With Type 2 Diabetes Treated With Sulfonylureas or SGLT2i Stratified by Variables

Variables	With sulfonylureas			With SGLT2i							
	N	PY	IR	N	PY	IR	cHR	95% CI	aHR[a]	95% CI	P for interaction
Sex											0.530
Female	172*	40731	4.22	116	41148	2.82	0.67	(0.53, 0.85)	0.65	(0.52, 0.83)	
Male	274	54839	5.00	168	55296	3.04	0.61	(0.5, 0.73)	0.58	(0.48, 0.71)	
Age											0.203
20-40	51	12211	4.18	25	12579	1.99	0.47	(0.29, 0.76)	0.47	(0.29, 0.75)	
41-60	230	47898	4.80	139	48653	2.86	0.59	(0.48, 0.73)	0.57	(0.46, 0.70)	
61-80	165	35462	4.65	120	35211	3.41	0.73	(0.58, 0.93)	0.72	(0.57, 0.91)	
Comorbidities											

© 2023 Yen F-S et al. *JAMA Network Open*

Obesity	9	6870	1.31	16	7537	2.12	1.61	(0.71, 3.64)	1.62	(0.71, 3.72)	0.021
Smoking	22	4876	4.51	6	4724	1.27	0.28	(0.11, 0.68)	0.29	(0.12, 0.71)	0.071
Hypertension	288	66156	4.35	192	66105	2.90	0.67	(0.55, 0.8)	0.64	(0.53, 0.77)	0.310
Dyslipidemia	269	73772	3.65	176	73415	2.40	0.66	(0.54, 0.8)	0.63	(0.52, 0.76)	0.427
Coronary artery disease	83	26781	3.10	76	26920	2.82	0.91	(0.67, 1.24)	0.89	(0.65, 1.21)	0.010
Stroke	37	9731	3.80	29	9613	3.02	0.80	(0.49, 1.31)	0.80	(0.49, 1.30)	0.324
Heart failure	17	5467	3.11	25	5741	4.35	1.40	(0.75, 2.59)	1.38	(0.74, 2.58)	0.009
Atrial fibrillation	29	10812	2.68	26	10633	2.45	0.95	(0.56, 1.63)	0.94	(0.55, 1.61)	0.161
Peripheral arterial disease											0.624
COPD	75	24484	3.06	61	24254	2.52	0.82	(0.58, 1.15)	0.79	(0.56, 1.11)	0.092
Liver cirrhosis	6	1377	4.36	8	1506	5.31	1.21	(0.42, 3.48)	1.20	(0.40, 3.62)	0.210
Chronic kidney disease	40	6992	5.72	28	6748	4.15	0.72	(0.45, 1.17)	0.72	(0.44, 1.17)	0.585

© 2023 Yen F-S et al. *JAMA Network Open*

Diabetic retinopathy	67	5837	11.48	61	6726	9.07	0.80	(0.56, 1.13)	0.86	(0.60, 1.23)	0.112
CCI											0.027
0	272	61707	4.41	162	61102	2.65	0.60	(0.49, 0.73)	0.59	(0.48, 0.71)	
1	81	17049	4.75	38	17585	2.16	0.46	(0.31, 0.67)	0.43	(0.29, 0.63)	
≥2	93	16815	5.53	84	17757	4.73	0.86	(0.64, 1.16)	0.85	(0.63, 1.15)	
DCSI											0.069
0	138	31304	4.41	68	31174	2.18	0.50	(0.37, 0.67)	0.50	(0.38, 0.67)	
1	99	18876	5.24	58	19104	3.04	0.58	(0.42, 0.8)	0.58	(0.42, 0.80)	
≥2	209	45391	4.60	158	46166	3.42	0.74	(0.6, 0.91)	0.70	(0.57, 0.87)	
Medications											
Metformin	365	83848	4.35	228	83786	2.72	0.63	(0.53, 0.74)	0.61	(0.52, 0.72)	0.900
Thiazolidinediones	47	9557	4.92	45	10877	4.14	0.86	(0.57, 1.29)	0.81	(0.54, 1.23)	0.122

© 2023 Yen F-S et al. *JAMA Network Open*

DPP-4i	187	36164	5.17	121	38886	3.11	0.61	(0.48, 0.76)	0.61	(0.48, 0.77)	0.682
Alpha-glucosidase inhibitors	72	12846	5.60	48	13920	3.45	0.63	(0.43, 0.9)	0.60	(0.42, 0.87)	0.933
Numbers of oral antidiabetic drugs											0.731
0-1	223	52741	4.23	135	51004	2.65	0.62	(0.5, 0.77)	0.59	(0.47, 0.73)	
2-3	209	40990	5.10	141	43086	3.27	0.65	(0.52, 0.8)	0.65	(0.53, 0.81)	
>3	14	1840	7.61	8	2354	3.40	0.47	(0.2, 1.13)	0.41	(0.17, 0.99)	
GLP-1 RA	61	11584	5.27	34	11898	2.86	0.55	(0.36, 0.84)	0.51	(0.33, 0.79)	0.449
Insulin	182	31486	5.78	156	33480	4.66	0.81	(0.65, 1)	0.76	(0.61, 0.94)	0.001
Statin	219	63448	3.45	160	63579	2.52	0.73	(0.59, 0.89)	0.69	(0.56, 0.85)	0.043
Aspirin	137	37664	3.64	104	37694	2.76	0.76	(0.59, 0.98)	0.73	(0.56, 0.94)	0.088

PY, Person-Year; IR, incidence rate, per 1000 person-years; cHR, crude hazard ratio; aHR, adjusted hazard ratio; CI, confidence interval; COPD, chronic obstructive pulmonary disease; SGLT2i, sodium-glucose cotransporter 2 inhibitor; DPP-4i, dipeptidyl peptidase-4 inhibitor; GLP-1 RA, glucagon-like peptide-1 receptor agonist; CCI, Charlson Comorbidity Index; DCSI, Diabetes Complications Severity Index; aHR[a]: adjusted for age, sex, obesity, smoking, Charlson Comorbidity Index, Diabetes Complications Severity Index score, comorbidities, medications, and duration

© 2023 Yen F-S et al. *JAMA Network Open*

of diabetes as shown in table 1 with the Cox proportional hazards regression.

* This means that during the 40731 patient-years of follow-up in the matched female sulfonylurea users, 172 women developed sight-threatening retinopathy, with an incident rate of 4.42 (per 1000 PY).

© 2023 Yen F-S et al. *JAMA Network Open*

eTable 5. Risk of Sight-threatening retinopathy associated with SGLT2i, DPP4i, pioglitazone and sulfonylureas Use

Variable	n	PY	IR	cHR	(95% CI)	P value	aHR[a]	(95% CI)	P value
DPP-4i	928	151416	6.13	1.00	(reference)	-	1.00	(reference)	-
Empagliflozin	45	12735	3.53	0.55	(0.41, 0.75)	<0.001	0.53	(0.4, 0.72)	<0.001
Dapagliflozin	319	96381	3.31	0.55	(0.48, 0.62)	<0.001	0.54	(0.48, 0.62)	<0.001
Canagliflozin	289	72214	4.00	0.66	(0.58, 0.75)	<0.001	0.65	(0.57, 0.74)	<0.001
Pioglitazone	1392	241470	5.76	1.00	(reference)	-	1.00	(reference)	-
Empagliflozin	59	17752	3.32	0.56	(0.43, 0.73)	<0.001	0.57	(0.44, 0.74)	<0.001

© 2023 Yen F-S et al. *JAMA Network Open*

Dapagliflozin	615	149005	4.13	0.72	(0.66, 0.79)	<0.001	0.73	(0.66, 0.8)	<0.001
Canagliflozin	602	127306	4.73	0.82	(0.75, 0.91)	<0.001	0.81	(0.74, 0.89)	<0.001
Sulfonylureas	446	95571	4.67	1.00	(reference)	-	1.00	(reference)	-
Empagliflozin	22	7271	3.03	0.62	(0.41, 0.96)*	0.031	0.6	(0.39, 0.92)	0.02
Dapagliflozin	163	57460	2.84	0.61	(0.51, 0.73)	<0.001	0.61	(0.51, 0.73)	<0.001
Canagliflozin	170	49830	3.41	0.74	(0.62, 0.88)	<0.001	0.71	(0.59, 0.85)	<0.001

PY, Person-Year; IR, incidence rate, per 1000 person-years; cHR, crude hazard ratio; aHR, adjusted hazard ratio; CI, confidence interval; SGLT2i, sodium-glucose cotransporter 2 inhibitor; DPP-4i, dipeptidyl peptidase-4 inhibitor; aHR[a]: adjusted for age, sex, obesity, smoking, Charlson Comorbidity Index, Diabetes Complications Severity Index score, comorbidities, medications, and duration of diabetes as shown in Table 1 with the Cox proportional hazards regression.

© 2023 Yen F-S et al. *JAMA Network Open*

eTable 6. Risk of Outcomes between SGLT2i, DPP-4i, pioglitazone and sulfonylurea Use

Variables	Dialysis								
	n	PY	IR	cHR	(95% CI)	P value	aHR[a]	(95% CI)	P value
DPP-4i	361	15240	2.37	1.00	(reference)		1.00	(reference)	
SGLT2i	16	154380	0.1	0.04	(0.03, 0.07)	<0.001	0.05	(0.03, 0.08)	<0.001
Pioglitazone	606	243042	2.49	1.00	(reference)		1.00	(reference)	
SGLT2i	95	244291	0.39	0.16	(0.13, 0.19)	<0.001	0.18	(0.14, 0.22)	<0.001
Sulfonylureas	125	96082	1.30	1.00	(reference)		1.00	(reference)	
SGLT2i	20	96806	0.21	0.16	(0.1, 0.25)	<0.001	0.16	(0.1, 0.25)	<0.001

© 2023 Yen F-S et al. *JAMA Network Open*

Variables	Hospitalization for heart failure								
	n	PY	IR	cHR	(95% CI)		aHR[a]	(95% CI)	
DPP-4i	892	151727	5.88	1.00	(reference)		1.00	(reference)	
SGLT2i	421	153836	2.74	0.47	(0.41, 0.52)	<0.001	0.47	(0.41, 0.52)	<0.001
Pioglitazone	1538	241878	6.36	1.00	(reference)	-	1.00	(reference)	-
SGLT2i	1214	242748	5.00	0.79	(0.73, 0.85)	<0.001	0.81	(0.75, 0.87)	<0.001
Sulfonylureas	460	95701	4.81	1.00	(reference)	-	1.00	(reference)	-
SGLT2i	312	96441	3.24	0.67	(0.58, 0.78)	<0.001	0.66	(0.57, 0.76)	<0.001

© 2023 Yen F-S et al. *JAMA Network Open*

Variables	Severe Hypoglycemia								
	n	PY	IR	cHR	(95% CI)		aHR[a]	(95% CI)	
DPP-4i	603	152047	3.97	1.00	(reference)	-	1.00	(reference)	-
SGLT2i	268	154035	1.74	0.44	(0.38, 0.51)	<0.001	0.44	(0.38, 0.51)	<0.001
Pioglitazone	1669	241228	6.92	1.00	(reference)	-	1.00	(reference)	-
SGLT2i	830	243261	3.41	0.49	(0.45, 0.54)	<0.001	0.5	(0.46, 0.54)	<0.001
Sulfonylureas	415	95666	4.34	1.00	(reference)	-	1.00	(reference)	-
SGLT2i	156	96613	1.61	0.37	(0.31, 0.45)	<0.001	0.35	(0.29, 0.42)	<0.001

PY, Person-Year; IR, incidence rate, per 1000 person-years; cHR, crude hazard ratio; aHR, adjusted hazard ratio; CI, confidence interval; COPD, chronic obstructive pulmonary disease; SGLT2i, sodium-glucose cotransporter 2 inhibitor; DPP-4i, dipeptidyl peptidase-4 inhibitor; GLP-1 RA,

© 2023 Yen F-S et al. *JAMA Network Open*

glucagon-like peptide-1 receptor agonist; aHR[a]: adjusted for age, sex, obesity, smoking, Charlson Comorbidity Index, Diabetes Complications Severity Index score, comorbidities, medications, and duration of diabetes as shown in Table 1 with the Cox proportional hazards regression.

© 2023 Yen F-S et al. *JAMA Network Open*

eFigure. Flowchart for the Selection of Matched Patients for SGLT2i, DPP-4i, Pioglitazone, and Sulfonylureas

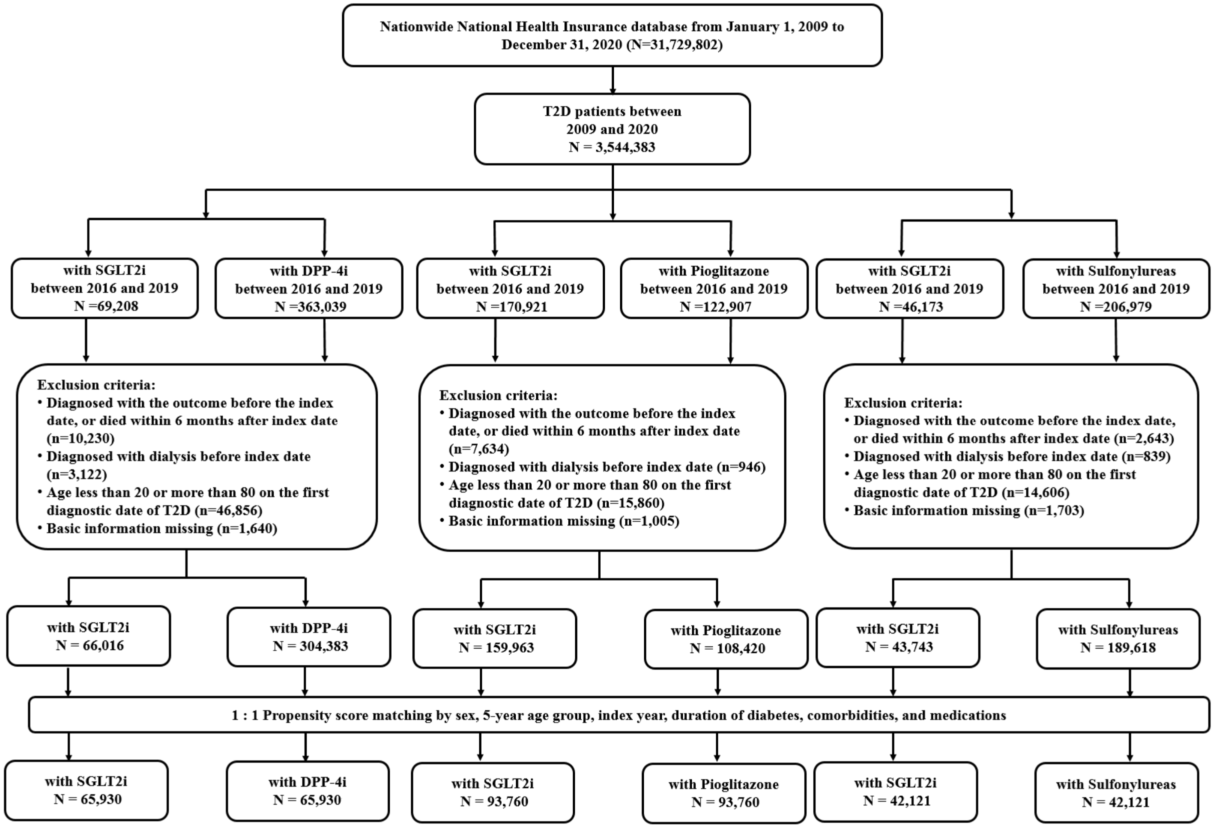

© 2023 Yen F-S et al. *JAMA Network Open*
