# Supplementary material for: Sodium-Glucose Cotransporter 2 Inhibitors and Risk of Retinopathy in Patients With Type 2 Diabetes
Source: JAMA Netw Open. 2023 Dec 20;6(12):e2348431. doi: 10.1001/jamanetworkopen.2023.48431 (PMC10733799; doi:10.1001/jamanetworkopen.2023.48431)
Supplement: Supplement 2. — Data Sharing Statement [file jamanetwopen-e2348431-s002.pdf]

Data Sharing Statement

Yen. Sodium-Glucose Cotransporter 2 Inhibitors and Risk of Retinopathy in Patients with Type 2 Diabetes. *JAMA Netw Open*. Published December 20, 2023.
doi:10.1001/jamanetworkopen.2023.48431

Data
Data available: No

Additional Information
Explanation for why data not available: Data of this study are available from the National Health Insurance Research Database (NHIRD) published by Taiwan National Health Insurance (NHI) Administration. The data utilized in this study cannot be made available in the paper, the supplemental files, or in a public repository due to the ‘‘Personal Information Protection Act’’ executed by Taiwan government starting from 2012. Requests for data can be sent as a formal proposal to the NHIRD Office (https://dep.mohw.gov.tw/DOS/cp-2516-3591-113.html) or by email to stsung@mohw.gov.tw.
